# Chronic Illness, Nutritional Status, and Factors Associated with Malnutrition among Various Age Groups Residing in Urban Areas of Telangana and Rural Areas of Andhra Pradesh

**DOI:** 10.3390/nu15204470

**Published:** 2023-10-22

**Authors:** Karthikeyan Ramanujam, Nagaraju Mergu, Henna Kondeth, Garlapati Venkat Raji Reddy, Upadrasta Venkata Prasad, Renuka Sadasivuni, Jagajeevan Babu Geddam, Hemalatha Rajkumar, Nusi Samarasimha Reddy

**Affiliations:** 1 Clinical Epidemiology Division, ICMR-National Institute of Nutrition, Hyderabad 500 007, Telangana, India; karthikjpt@gmail.com (K.R.); nagarajumergu30@gmail.com (N.M.); gvenkatrajireddy1980@gmail.com (G.V.R.R.); geddambabuj@gmail.com (J.B.G.); 2 Jawaharlal Institute of Postgraduate Medical Education and Research, Puducherry 605 006, Puducherry, India; hennakondeth1210@gmail.com; 3 Model Rural Health Research Unit, ICMR-National Institute of Nutrition, Chandragiri 517 101, Andhra Pradesh, India; prasaduv@gmail.com (U.V.P.); renu1210@gmail.com (R.S.); 4 ICMR-National Institute of Nutrition, Hyderabad 500 007, Telangana, India; rhemalathanin@gmail.com

**Keywords:** malnutrition, stunting, obesity, chronic diseases, risk factors, rural and urban India

## Abstract

Malnutrition includes both under-nutrition and over-nutrition, which have negative health impacts and social consequences. The present study aims to understand the demographic dynamics, burden of chronic illnesses, and risk factors associated with malnutrition (stunting, thinness, and obesity) among different age groups in urban and rural areas. Data were collected through a cross-sectional study conducted in an urban area in Hyderabad and four rural villages in Andhra Pradesh. A multivariable mixed-effect logistic regression was used to assess the risk factors associated with malnutrition among different age groups. The final analysis included the data of 10,350 individuals, consisting of 8317 (80.4%) from urban areas and 2033 (19.6%) from rural areas. The number of known cases of hypertension in the urban area was 926 (11.1%) and 114 (5.6%) in the rural areas, and that of diabetes was 511 (6.1%) in the urban area and 104 (5.1%) in the rural areas. The burden of stunting among under-five children and obesity among adults was 33.7% (95% CI; 29.7–37.9) and 47.4% (95% CI; 45.8–49.1), respectively. Adults aged 40–59 years (AOR 1.91; 1.59–2.28) and belonging to a clerical/skilled (AOR 1.32; 1.03–1.71) occupation were at higher odds of obesity compared to their counterparts. Policymakers and health practitioners should consider the insights from our findings to tailor effective interventions to address malnutrition.

## 1. Introduction

Malnutrition is a global public health problem affecting people of all ages and in all parts of the world [1]. Malnutrition includes both under-nutrition and over-nutrition, which have negative health impacts and social consequences [2]. According to the WHO, malnutrition accounts for nearly 45% of all deaths among under-five children globally [1]. According to the Global Nutrition Report 2020, roughly one in every three people worldwide suffers from at least one form of malnutrition, including under-nutrition, micronutrient deficiencies, and overweight/obesity [3]. Moreover, malnutrition affects a person’s quality of life and productivity throughout their lifetime through having long lasting impacts on their physical and cognitive development [2].

Underweight is more common than overweight and obesity in developing countries compared to developed countries, although it is predicted that soon they will overtake the rate of obesity in developed countries [4,5]. Because developing countries are seeing significant changes in terms of activity and diet patterns, a move from active to sedentary behaviours [5,6], they are facing a “double burden of malnutrition”, meaning both underweight and overweight [7]. Among older individuals, whereas being underweight causes exhaustion and persistent weakness, an increased chance of infection, and mortality, being overweight causes more chronic non-communicable illnesses such as diabetes, coronary artery disease, hypertension, weakened functioning, disability, and mortality [8]. In low- and middle-income countries, the coexistence of under nutrition with an increasing incidence of overweight/obesity is a serious health concern [9,10,11].

Obesity and overweight pose a significant burden on both adults and adolescents, affecting their physical, emotional, and social well-being. In adults, these conditions have reached epidemic proportions and are associated with a plethora of health issues [12]. A study conducted by the Global Burden of Disease (GBD) 2017 Diet Collaborators reported that high body mass index (BMI) was the fourth leading risk factor for mortality globally [13]. Moreover, obesity-related healthcare costs have been escalating, straining healthcare systems and economies [14]. Similarly, the prevalence of obesity and overweight among adolescents has risen dramatically in recent years. This trend poses serious immediate and long-term health consequences, such as insulin resistance, metabolic syndrome, and mental health problems such as depression and anxiety [15]. Obese or overweight adolescents are more likely to carry these conditions into adulthood, further increasing their risk of developing chronic diseases [16].

Even though the country continues to see an increase in overweight/obesity, India has the largest percentage of underweight adults globally [17,18]. According to the National Family Health Survey (NFHS-5) conducted in 2019–21, among children under five years of age, 35.5% were stunted, 32.1% were underweight, and 19.3% had wasting. The same survey showed that 19% of women and 16% of men aged 15–49 years were undernourished, while 24% of women and 23% of men aged 15–49 years were obese [19]. Overweight and obesity are well-established risk factors for general mortality [20], chronic illnesses, including cardiovascular disease [21], diabetes [22], multimorbidity [23,24], and impairments [25]. Furthermore, being underweight is highly connected with early mortality, disability, and poor self-evaluation of health and wellness, and this relationship is especially strong in developing countries [26,27]. Studies have revealed that socioeconomic variables such as poverty, poor household income, and inadequate access to high-quality healthcare might affect the incidence of malnutrition differently in rural and urban parts of India [1,28]. Additionally, dietary patterns [1], sanitation and hygiene [28], and maternal and child health [1,3] are also important to maintain a good nutritional status.

The goal is to develop and implement targeted interventions and strategies that will effectively address malnutrition and its associated health issues, with a focus on improving the overall health and well-being of individuals in urban and rural communities. The data contribution to the existing knowledge on malnutrition assists in the formulation of evidence-based public health policies both in India and potentially in other regions facing similar challenges. The present study aims to understand the demographic dynamics, burden of non-communicable diseases, and risk factors associated with malnutrition (stunting, thinness, and obesity) among different age groups in the urban and rural areas.

## 2. Materials and Methods

### 2.1. Study Design and Setting

A cross-sectional study was conducted in Addagutta (17.4506° N, 78.5114° E), an urban area in Hyderabad city, Telangana state, and four rural villages (Rayalapuram (13.5791° N, 79.3155° E), Dornakambala (13.5467° N, 79.3045° E), Narasingapuram (13.6182° N, 79.2979° E), and Mittapalem (13.6074° N, 79.3235° E)) in Chandragiri mandal, Chittoor district of Andhra Pradesh state within the field practice area of Model Rural Health Research Unit (MRHRU) (13.5881° N, 79.3163° E) from March 2022 to January 2023. The households and individuals residing in the selected area were recruited for the study. Data were collected from all the residents of the households available at the time of the survey.

Data were collected using electronic tablets in the form of electronic forms. The questionnaire in the electronic forms was available in English and the local language (Telugu). Most of the responses were selected through a drop-down menu and entered in English. The tools used for the anthropometry included digital weighing scale and a stadiometer. Heights were measured using a stadiometer, with participants positioned against a flat surface to ensure heels, shoulders, and head were in contact with the wall. The stadiometer movable headpiece was gently lowered to record centimetre-accurate height measurements. Weights were taken on digital scales after removing footwear and socks to ensure precision. Trained investigators meticulously adhered to defined protocols throughout the data collection. Height measurements were obtained using a SECA adult portable stadiometer with 0.1 cm accuracy, while weight assessment utilized calibrated SECA digital weighing scales, documenting weights in kilograms with a precision of 0.1 kg.

### 2.2. Study Variables

We utilized a comprehensive set of demographic and socioeconomic variables to understand the intricate dynamics of the population. These variables include gender (male, female, transgender), religion (Hindu, Muslim, Christianity, others), house ownership (own, rented/leased/others), family type (nuclear, extended/three-generation family, joint), type of house (Pucca, mixed, Kutcha), overcrowding status (not overcrowded, overcrowded), place of cooking (separate kitchen, no separate kitchen), cooking fuel source (gas, firewood/others), water source (piped water, public tap/well/bore), defecation method (home toilet, public/shared toilet, open field), garbage disposal method (garbage services, drainage, no designated place), occupation (professional, skilled, unskilled worker, unemployed, student), literacy (age >7 years) (illiterate, read, read and write, preschool/Balwadi school), and wealth index (lowest, middle, highest). Overcrowding is defined as the number of persons living in the house divided by the number of rooms in the house, excluding the kitchen. It was categorized as overcrowded if there were more than two people per room and not overcrowded when there were ≤ two persons per room [29]. The wealth index was calculated by categorizing respondents into tertiles, i.e., lowest, middle, and highest wealth, based on their ownership of 28 assets. Data were collected on the burden of chronic illness using a questionnaire. The WHO Child Growth Standards were used for the determination of nutritional status of under-5 children. The standard deviation of scores (Z-scores) for height-for-age, weight-for-height, and weight-for-age were calculated using WHO Anthro software (version 3.2.2). Furthermore, the malnutrition cutoffs are categorized as stunting when height-for-age < −2 SD, wasting when weight-for-height < −2 SD, overweight when weight-for-height > +2 SD, and underweight when weight-for-age < −2 standard deviations (SD) of the WHO Child Growth Standards median [30]. Z–Scores for height-for-age and BMI-for-age were calculated for the 5–19-year group using WHO Anthro Plussoftware (version 1.0.4). In addition, the cut-offs of malnutrition status were categorised as height-for-age < −2 SD, considered stunting and BMI-for-age (BAZ) < −2 SD, BAZ > 1 SD, and BAZ > 2 SD, defined as thinness, overweight, and obesity, respectively [31]. The age group greater than 19 and less than 60 was considered the adult category. Furthermore, the age group 60 and above was considered the elderly (or geriatric) group [32]. Body mass index (BMI) was calculated for all the available heights and weights, and the calculation is stated as BMI (Kg/m^2^) = Weight (Kg)/Height (m^2^). These BMI values are categorised as underweight when BMI < 18.5 Kg/m^2^, normal when BMI is 18.5–22.9 Kg/m^2^, overweight when BMI is 23–24.9 Kg/m^2^, and obese when BMI ≥ 25 Kg/m^2^ [33].

### 2.3. Ethics Approval and Consent

The study protocol for this research was approved by the ICMR–National Institute of Nutrition Ethics Committee (reference number: 3/II/2022; dated 16 March 2022). Following the approval, data collection commenced, and consent forms were distributed to potential study participants in the local language (Telugu). Only individuals who willingly provided written informed consent were included in the study, while those who declined were excluded.

### 2.4. Statistical Analysis

Variables were summarized as frequencies and proportions with 95% confidence intervals. A univariate mixed-effect logistic regression was applied to assess stunting in under-5 children, thinness in the 5–19-year age group, and obesity in adult age groups, and the results were presented as an unadjusted odds ratio with a 95% confidence interval. The variables that had *p* value less than 0.25 in the univariate analysis and variables of clinical or contextual importance were included in the multivariable mixed-effect logistic regression to assess risk factors associated with malnutrition status. The results were presented as an adjusted odds ratios with a 95% confidence interval. The significance of the odds ratio was tested by Wald test. All the statistical analysis was carried out using STATA 14.1 version.

### Mixed-Effect Logistic Regression

The data exhibit a clustered structure, with multiple individuals nested within each household. Neglecting this interdependency may introduce biased standard errors and lead to incorrect inferences, as it violates the assumption of independence in a simple logistic regression [34]. In order to address the clustered structure of the data and to properly account for the correlation among individuals within households, we employed a mixed-effects logistic regression (MELR). This approach allowed us to model random effects associated with households, capturing the unobserved heterogeneity at the household level. We adjusted the analysis at the family level by utilizing a unique family identification number as a random effect in the MELR model, which accounted for unobserved factors that may influence the outcome variables but were not directly measured as predictor variables. To quantify the extent of clustering within households, we calculated the intraclass correlation coefficient (ICC), which revealed values greater than 0.4 for all outcome variables, indicating substantial clustering within households. This finding further supported the use of a MELR to properly account for the correlated nature of the data. Additionally, we assessed the Akaike Information Criterion (AIC) and the Bayesian Information Criterion (BIC) for both simple logistic regression and MELR models. Notably, the MELR models exhibited smaller information criteria values compared to simple logistic regression, indicating improved model fit [34]. The AIC and BIC were utilized to compare models, and the mixed-effect logistic regression model demonstrated the best fit for the data as it had the lowest values among the considered models (Supplementary Table S1). In the univariate analysis, variables with *p* value less than 0.25 and variables that are of clinical or contextual importance were included in a multivariable mixed-effect logistic regression (Supplementary Table S2). The variables place of residence, age group, gender, religion, house ownership, type of family, type of house, overcrowding, place of cooking, cooking fuel, source of water, method of defecation, occupation, and wealth index were included.

## 3. Results

### 3.1. General Characteristics of the Respondents

The final analysis included the data of 10,350 individuals, consisting of 8317 (80.4%) from urban areas and 2033 (19.6%) from rural areas. Among the 3258 households that were surveyed, 484 (14.9%) had temporary door locks, while 33 (1.0%) households refused to give their consent, and the final analysis included the data of 2741 (84.1%) households. The gender distribution was almost equal, with males accounting for 5126 (49.6%) and females for 5221 (50.4%). In terms of religion, Hindus comprised the majority at 8567 (82.8%), followed by Muslims at 1193 (11.5%) and Christians at 574 (5.5%). In total, 6839 (66.1%) individuals belonged to nuclear families, 4651 (55.9%) individuals were residing in overcrowded households in urban areas, and 9302 (89.9%) individuals used piped water for drinking and cooking. The respondents wealth index categories were “Lowest”, 4469 (43.2%), ”Middle”, 2434 (23.5%), and “Highest”, 3447 (33.3%) (Table 1).

**Figure 1:**
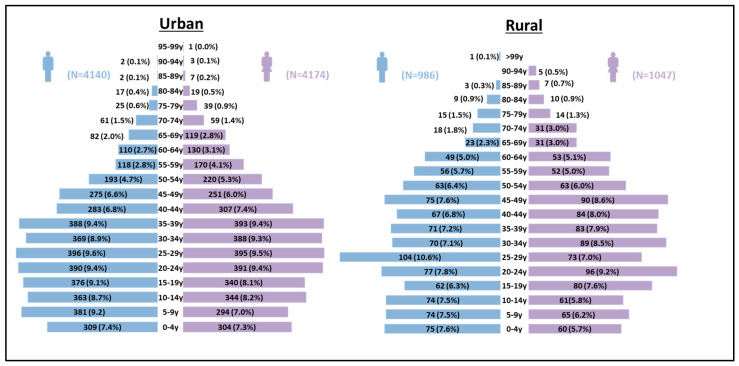
Age pyramid of the study population using the data from urban (*n* = 8317) area in Telangana and rural areas (*n* = 2033) in Andhra Pradesh. Three transgender people were excluded from building age pyramid.

The sample encompassed individuals ranging from 0 to 99 years of age. In the younger age groups (0–4 years, 5–9 years, 10–14 years, and 15–19 years), the number of males and females was relatively similar in both the urban and rural areas. The 20–24-year age group showed nearly equal representation of males and females, accounting for 9.4% in the urban population and 7.8% in the rural population. Similar patterns were observed in subsequent age groups, with slight variations in the proportions (Figure 1) (Section 2).

### 3.2. Malnutrition Status

Among under-five children, the prevalence of stunting was 33.8% (95%CI: 29.4–38.5; 143/423) in urban areas and 33.0% (95% CI: 24.1–43.3; 31/94) in rural areas. The prevalence of overweight was 4.0% (95% CI: 2.5–6.4; 17/423) among urban children and 2.1% (95% CI: 0.5–8.3; 2/94) among rural children. In the 5–19-year age group, thinness was seen among 31.7% (95% CI: 28.6–35.0; 254/801) of urban individuals and 15.9% (95% CI: 10.7–22.8; 23/145) of rural individuals. Obesity was found in 4.4% (95% CI: 3.2–6.0; 35/801) of urban individuals and 6.9% (95% CI: 3.7–12.4; 10/145) of rural individuals. Among adults in urban areas, obesity was seen among 47.7% (95% CI: 45.7–49.6; 1234/2590) of individuals and overweight among 14.8% (95% CI: 13.5–16.3; 384/2590) of individuals. Among adults in rural areas, obesity was seen among 46.7% (95% CI: 43.4–50.1; 396/847) of individuals and overweight among 14.8% (95% CI: 12.5–17.3; 125/847) of individuals. Among adults, underweight was seen in 11.6% (95% CI: 10.4–12.9; 301/2590) of individuals in urban areas and 12.3% (95% CI: 10.2–14.7; 104/847) of individuals in rural areas. In the geriatric age group, 50.6% (95% CI.: 46.0–55.1; 235/465) of urban individuals and 33.2% (95% CI: 27.2–39.8; 72/217) of rural individuals were found to be obese. In the geriatric age group, the proportion of underweight individuals in urban areas was 7.7% (95% CI: 5.6–10.6; 36/465), and in rural areas, it was 21.2% (95% CI: 16.2–27.2; 46/217) (Table 2).

### 3.3. Chronic Illness among Study Participants

**Figure 2:**
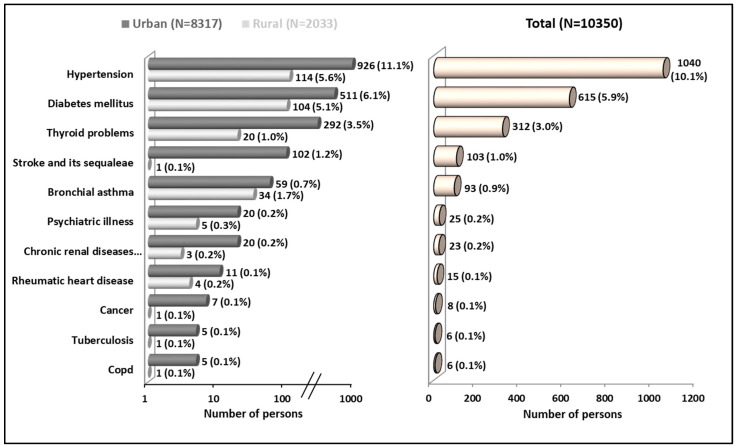
Burden of chronic illness using the data from urban and rural areas in South India.

Hypertension was the most prevalent chronic illness, affecting 1040 (10.1%) individuals, followed by diabetes mellitus, affecting 615 (5.9%), thyroid problems affecting 312 (3.0%), stroke and its sequelae affecting 103 (1.0%), and bronchial asthma affecting 93 (0.9%) (Figure 2).

### 3.4. Factors Associated with Stunting among Under-Five Children

It was found that 0–2-year-old children were at higher odds of being stunted compared to 3–5-year-old children (AOR 2.32, 95% CI: 1.29–4.18). Children living in mixed house were at higher odds of being stunted compared to children living in Pucca houses (AOR 2.48, 95% CI: 1.12–5.47). Children from the middle (AOR 3.47, 95% CI: 1.27–9.45) and lower wealth index tertiles (AOR 3.46, 95% CI: 1.32–9.06) were at higher odds of being stunted compared to children from the high wealth index tertiles (Table 3).

### 3.5. Factors Associated with Thinness among Adolescents

Urban residents were at higher odds of thinness compared to rural residents (AOR 2.52, 95% CI: 1.10–5.75). Males were at higher odds of thinness compared to females (AOR 2.62, 95% CI: 1.75–3.93). People living in overcrowded houses were at higher odds of being thin compared to people living in non-overcrowded houses (AOR 1.68, 95% CI: 1.04–2.71) (Table 3).

### 3.6. Factors Associated with Malnutrition among Adults

It was found that 40–59-year-old adults were at higher odds of being obese compared to 20–39-year-old adults (AOR 1.91, 95% CI: 1.59–2.28). Males were at decreased odds of being obese compared to females (AOR 0.79, 95% CI: 0.66–0.93). Individuals from muslim religion were at higher odds of being obese compared to individuals from hindu religion (AOR 1.73, 95% CI: 1.28–2.34). Individuals with an occupation as a clerk/skilled worker/semi-skilled worker had higher odds of being obese compared to unskilled workers (AOR 1.32, 95% CI: 1.02–1.71). Individuals from the low wealth index tertiles were at decreased odds of developing obesity compared to individuals from the high wealth index tertiles (AOR 0.59, 95% CI: 0.46–0.74) (Table 3).

## 4. Discussion

The findings highlight the recent demographic composition, burden of chronic illnesses, and malnutrition status among different age groups residing in the urban and rural areas of South India. Among children under five years of age, the prevalence of stunting and underweight remains high, despite some improvements over the years. The study reveals a high prevalence of obesity among adults in both urban and rural areas. Among under-five children, living in mixed houses and belonging to middle or lower wealth index tertiles were associated with higher odds of stunting. Among the 5–19-year age group, residing in urban areas, being male, and living in overcrowded conditions were associated with higher odds of thinness. In adults in the 40–59 age groups, belonging to the Muslim community and having a clerk or skilled worker occupation were associated with higher odds of obesity.

Stunting remains a significant public health concern, affecting approximately one-third of children in both urban and rural areas, almost similar to the NFHS 5 data of Telangana and Andhra Pradesh [35,36]. According to the Comprehensive National Nutrition Survey report (2016–2018), the proportion stunting in under-five children is 37% in rural and 27% in urban areas, which is in contrast to our findings of 33% in rural and 34% in urban areas [37]. The reason for these differences is that our study was conducted in selected areas in two states with a small sample size compared to the CNNS study. Children from the lowest wealth quintile have a higher likelihood of being stunted, similar to the findings of the Comprehensive National Nutrition Survey report (2016–2018) [37]. The age-specific analysis indicates that children between 0 and 2 years are particularly vulnerable to stunting.

Overweight and obesity are emerging as major health challenges in both urban and rural settings. This study reported the prevalence of overweight and obesity in adults as 14.8% and 47.7%, respectively, in contrast to the findings from the studies conducted in Karnataka [38] and Assam [39]. In the urban geriatric population, more than 50% of the population were found to be obese. This shift towards over-nutrition could be attributed to changes in lifestyle, including dietary patterns and sedentary behaviour, as well as the lack of comprehensive strategies to promote healthy eating habits and physical activity. The prevalence of obesity is highest in the 40–59 age group, indicating that middle-aged adults are at the greatest risk. Gender disparities are also observed, with females having higher odds of thinness and males having higher odds of obesity. These findings highlight the importance of gender-sensitive interventions to address malnutrition in different age groups.

The high prevalence of overweight and obesity leads to related morbidities, notably hypertension and diabetes mellitus [40], which are also found in our study population. These findings are consistent with the epidemiological transition occurring in many developing regions, where non-communicable diseases are on the rise, accompanied by the persistent challenges of infectious diseases and malnutrition [41]. The prevalence of chronic illness underscores the importance of adopting a holistic approach to healthcare that encompasses the prevention, early detection, and effective management of these conditions.

The study identified several risk factors associated with malnutrition and chronic illnesses. Socio-demographic factors such as place of residence, house ownership, and family type were found to be significant predictors of malnutrition. Overcrowding and poor housing conditions in rural areas were associated with increased odds of malnutrition, indicating the need for improved living conditions and access to basic amenities. Age, gender, religion, house ownership, occupation, and wealth index were significantly associated with obesity in adults.

This study utilized a rigorous methodology with a large sample size, enhancing the generalizability of the findings. The use of electronic data collection tools facilitated efficient data collection and minimized the errors associated with paper-based surveys. This study establishes the fact of the double burden of malnutrition in different age groups in India, along with the associated risk factors from more recent data. However, some limitations should be acknowledged. The cross-sectional design of this study limits its ability to establish causal relationships between risk factors and health outcomes; self-reported data on chronic illnesses may introduce reporting bias; and the prevalence of chronic illnesses could be underestimated.

The findings of this study have significant implications for public health policy and interventions. Targeted nutrition programs aimed at reducing malnutrition should prioritize interventions during the critical periods of early childhood and adolescence. These efforts should include nutrition education for caregivers and communities to promote optimal feeding practices. To tackle the rising burden of chronic illnesses, comprehensive prevention and management strategies are essential. Public health initiatives should focus on promoting awareness about risk factors for chronic illnesses. Integrating non-communicable disease management into the primary healthcare system can improve early detection and access to essential services. Following the population over a period by establishing the health and demographic surveillance system will help us understand the modifiable risk factors for planning appropriate interventions in this population.

## 5. Conclusions

Our cross-sectional study included a diverse population across various age groups residing in both urban and rural areas: under-five children, adolescents (10–19 years), adults (20–59 years), and geriatric individuals (60 years and above). We found significant associations between socio-demographic factors and malnutrition indicators among the different age groups. Particularly, there was a considerable prevalence of stunting among under-five children, especially in urban areas. In the 5–19-year age group, both urban residents and males showed higher susceptibility to thinness. In adults, obesity was notably prevalent, especially among middle-aged individuals, males, and those with specific household characteristics. Our findings highlight the importance of targeted interventions to address malnutrition across diverse populations. Public health strategies should focus on improving living conditions, promoting healthier cooking practices, and fostering awareness about balanced nutrition. Policymakers and health practitioners must consider these insights to tailor effective interventions that address the multifaceted factors contributing to malnutrition within distinct age groups and residential settings.

## Figures and Tables

**Table 1:** Socio-demographic, socio-economic, and individual-level characteristics of study participants using the data from urban (*n* = 8317) and rural areas (*n* = 2033) in South India.

Variable	Category	Urban (*N* = 8317) *n* (%)	Rural (*N* = 2033) *n* (%)	Total (*N* = 10,350) *n* (%)
Gender	Male	4140 (49.8)	986 (48.5)	5126 (49.6)
	Female	4174 (50.2)	1047 (51.5)	5221 (50.4)
	Transgender	3 (0.0)	–	3 (0.0)
Religion	Hindu	6617 (79.6)	1950 (95.9)	8567 (82.8)
	Muslim	1120 (13.5)	73 (3.6)	1193 (11.5)
	Christianity	564 (6.7)	10 (0.5)	574 (5.5)
	Others	16 (0.2)	–	16 (0.2)
	General	219 (2.6)	817 (40.2)	1036 (10.0)
House ownership	Own	5366 (64.5)	1790 (88.1)	7156 (69.1)
	Rented/leased/others	2951 (35.5)	243 (11.9)	3194 (30.9)
Type of family	Nuclear	5646 (67.9)	1193 (58.7)	6839 (66.1)
	Extended/three-generation family	2280 (27.4)	596 (29.3)	2876 (27.8)
	Joint	391 (4.7)	244 (12.0)	635 (6.1)
Type of house	Pucca	5495 (66.1)	1547 (76.1)	7042 (68.0)
	Mixed	2772 (33.3)	281 (13.8)	3053 (29.5)
	Kutcha	50 (0.6)	205 (10.1)	255 (2.5)
Overcrowding	Not overcrowded	3666 (44.1)	1175 (57.8)	4841 (46.8)
	Overcrowded	4651 (55.9)	858 (42.2)	5509 (53.2)
Place of cooking	Separate kitchen	4939 (59.4)	240 (11.8)	5179 (50.1)
	No separate kitchen	3378 (40.6)	1793 (88.2)	5171 (49.9)
Cooking fuel	Gas	8307 (99.9)	1742 (85.7)	10,049 (97.1)
	Firewood/others	10 (0.1)	291 (14.3)	301 (2.9)
Source of water for drinking and cooking	Piped water into residence/buying water with cans/water purifier in the house	8273 (99.5)	1029 (50.6)	9302 (89.9)
	Public tap/public well/bore/well on residence/plot	44 (0.5)	1004 (49.4)	1048 (10.1)
Most common method of defecation	Use toilet at home	8025 (96.5)	1602 (78.8)	9627 (93.0)
	Public/community/shared toilet	232 (2.8)	6 (0.3)	238 (2.3)
	Open field	60 (0.7)	425 (20.9)	485 (4.7)
Method of garbage disposal	Panchayat/corporation garbage disposal services/garbage dump/public pits	8208 (98.7)	725 (35.7)	8933 (86.3)
	Drainage/vacant/ abandoned house/burning	2 (0.0)	781 (38.4)	783 (7.6)
	No designated place	107 (1.3)	527 (25.9)	634 (6.1)
Occupation code	Professional/semi-professional	157 (1.9)	71 (3.5)	228 (2.2)
	Clerk/skilled workers/semi-skilled workers	3932 (47.3)	910 (44.8)	4842 (46.8)
	Unskilled worker	832 (9.9)	292 (14.4)	1124 (10.8)
	Unemployed	189 (2.3)	39 (1.8)	228 (2.2)
	Student/preschool/Balwadi school	3207 (38.6)	721 (35.5)	3928 (38)
Literate (age > 7 years) *	Illiterate	1601 (21.6)	446 (24.2)	2047 (22.0)
	Read	16 (0.2)	30 (1.6)	46 (0.5)
	Read and write	5817 (78.1)	1370 (74.1)	7187 (77.4)
	Preschool/Balwadi school	10 (0.1)	1 (0.1)	11 (0.1)
Wealth Index	Lowest	3649 (43.9)	820 (40.3)	4469 (43.2)
	Middle	2041 (24.5)	393 (19.4)	2434 (23.5)
	Highest	2627 (31.6)	820 (40.3)	3447 (33.3)

* Data were not collected for below 7 years; therefore, it does not sum up to *N*.

**Table 2:** Malnutrition status of study participants using the data from urban and rural areas in South India.

Malnutrition Status		Urban		Rural		Total	
		*N* = 8317		*N* = 2033		*N* =10,350	
		Proportion	% Prevalence	Proportion	% Prevalence	Proportion	% Prevalence
		(n/N)	(95% CI)	(n/N)	(95% CI)	(n/N)	(95% CI)
Under-5 Children	Stunting	143/423	33.8 (29.4–38.5)	31/94	33.0 (24.1–43.3)	174/517	33.7 (29.7–37.9)
	Wasting	83/423	19.6 (16.1–23.7)	20/94	21.3 (14.1–30.9)	103/517	19.9 (16.7–23.6)
	Overweight	17/423	4.0 (2.5–6.4)	2/94	2.1 (0.5–8.3)	19/517	3.7 (2.4–5.7)
	Underweight	138/438	31.5 (27.3–36.0)	30/96	31.3 (22.7–41.4)	168/534	31.5 (27.6–35.5)
5–19 Years	Stunting	131/802	16.3 (13.9–19.1)	25/145	17.2 (11.9–24.4)	156/947	16.5 (14.2–19)
	Thinness	254/801	31.7 (28.6–35.0)	23/145	15.9 (10.7–22.8)	277/946	29.3 (26.5–32.3)
	Overweight	95/801	11.9 (9.8–14.3)	24/145	16.6 (11.3–23.6)	119/946	12.6 (10.6–14.9)
	Obese	35/801	4.4 (3.2–6.0)	10/145	6.9 (3.7–12.4)	45/946	4.8 (3.6–6.3)
Adults (19–59 years)	Underweight (<18.5 Kg/m^2^)	301/2590	11.6 (10.4–12.9)	104/847	12.3 (10.2–14.7)	405/3437	11.8 (10.7–12.9)
	Normal (18.5–22.9 Kg/m^2^)	671/2590	25.9 (24.3–27.6)	222/847	26.2 (23.4–29.9)	893/3437	26.0 (24.5–27.5)
	Overweight (23–24.9 Kg/m^2^)	384/2590	14.8 (13.5–16.3)	125/847	14.8 (12.5–17.3)	509/3437	14.8 (13.7–16)
	Obese (≥25 Kg/m^2^)	1234/2590	47.7 (45.7–49.6)	396/847	46.7 (43.4–50.1)	1630/3437	47.4 (45.8–49.1)
Geriatric (60 years and above)	Underweight (<18.5 Kg/m^2^)	36/465	7.7 (5.6–10.6)	46/217	21.2 (16.2–27.2)	82/682	12.0 (9.8–14.7)
	Normal (18.5–22.9 Kg/m^2^)	126/465	27.1 (23.2–31.3)	71/217	32.7 (26.8–39.3)	197/682	28.9 (25.6–32.4)
	Overweight (23–24.9 Kg/m^2^)	68/465	14.6 (11.7–18.2)	28/217	12.9 (9.0–18.1)	96/682	14.1 (11.7–16.9)
	Obese (≥25 Kg/m^2^)	235/465	50.6 (46.0–55.1)	72/217	33.2 (27.2–39.8)	307/682	45 (41.3–48.8)

**Table 3:** Factors associated with stunting among under-five children, thinness among the 5–19-year-old group, and obesity among adults (19–59 years) through a multivariable mixed-effect logistic regression using the data from urban and rural areas in South India.

Variable	Category	Under-5 Children		5–19 Years		Adult (19–59 Years)	
		Stunting (Yes/No = 174/343)		Thinness (Yes/No = 277/669)		Obese (Yes/No = 1630/1807)	
		Adjusted OR (95% CI)	*p*–Value	Adjusted OR (95% CI)	*p*–Value	Adjusted OR (95% CI)	*p*–Value
Place of residence	Urban	1.14 (0.43–2.98)	0.795	2.52 (1.10–5.75)	0.028 *	0.84 (0.61–1.16)	0.290
	Rural	Ref	–	Ref	–	Ref	–
Age group in years	0–2	2.32 (1.29–4.18)	0.005 *	–	–	–	–
	3–5	Ref	–	–	–	–	–
	5–9	–		Ref	–	–	–
	10–14	–		1.13 (0.68–1.86)	0.639	–	–
	15–19	–		1.01 (0.61–1.66)	0.974	–	–
	20–39	–		–	–	Ref	–
	40–59	–		–	–	1.91 (1.59–2.28)	<0.001 *
Gender	Male	1.54 (0.85–2.80)	0.156	2.62 (1.75–3.93)	<0.001 *	0.79 (0.66–0.93)	0.006 *
	Female	Ref	–	Ref	–	Ref	–
Religion	Hindu	Ref	–	Ref	–	Ref	–
	Muslim	0.45 (0.17–1.22)	0.118	1.16 (0.60–2.26)	0.659	1.73 (1.28–2.34)	<0.001 *
	Christianity	1.63 (0.52–5.13)	0.407	0.85 (0.34–2.09)	0.719	1.17 (0.79–1.72)	0.435
House ownership	Own	Ref	–	Ref	–	Ref	–
	Rented/leased/others	1.40 (0.66–2.95)	0.383	0.96 (0.60–1.55)	0.876	0.78 (0.63–0.97)	0.023 *
Type of family	Nuclear	Ref	–	–	–	Ref	–
	Extended/three-generation family	0.82 (0.39–1.72)	0.594	–	–	0.91 (0.73–1.14)	0.415
	Joint	1.16 (0.35–3.92)	0.806	–	–	1.16 (0.78–1.73)	0.476
Type of house	Pucca	Ref	–	Ref	–	Ref	–
	Mixed	2.48 (1.12–5.47)	0.024 *	0.86 (0.54–1.36)	0.513	0.85 (0.69–1.05)	0.129
	Kutcha	2.91 (0.27–31.88)	0.382	0.61 (0.12–3.10)	0.547	0.76 (0.41–1.42)	0.390
Overcrowding	Not overcrowded	Ref	–	Ref	–	Ref	–
	Overcrowded	1.17 (0.58–2.37)	0.656	1.68 (1.04–2.71)	0.035 *	0.83 (0.68–0.99)	0.048 *
Place of cooking	Separate kitchen	Ref	–	–	–	Ref	–
	No separate kitchen	1.00 (0.51–1.95)	0.992	–	–	0.80 (0.66–0.98)	0.033 *
Cooking fuel	Gas	Ref	–	–	–	Ref	–
	Firewood/others	1.50 (0.15–14.83)	0.731	–	–	0.41 (0.23–0.74)	0.003 *
Source of water for drinking and cooking	Piped water into residence/buying water with cans/water purifier in the house	–	–	Ref	–	Ref	–
	Public tap /public well/bore/well on residence/plot	–	–	0.87 (0.27–2.78)	0.809	0.79 (0.56–1.13)	0.195
Most common method of defecation	Use toilet at home	–	–	–	–	Ref	–
	Public/community/shared Toilet	–	–	–	–	1.05 (0.59–1.88)	0.859
	Open field	–	–	–	–	0.97 (0.56–1.66)	0.900
Occupation code	Professional/semi- professional	–	–	–	–	1.14 (0.65–2.01)	0.649
	Clerk/skilled workers/semi-skilled workers	–	–	–	–	1.32 (1.02–1.71)	0.034 *
	Unskilled worker	–	–	–	–	Ref	–
	Unemployed	–	–	–	–	0.81 (0.49–1.35)	0.416
	Student	–	–	–	–	0.49 (0.33–0.73)	<0.001 *
Wealth Index	Lowest	3.46 (1.32–9.06)	0.012 *	1.34 (0.77–2.34)	0.294	0.59 (0.46–0.74)	<0.001 *
	Middle	3.47 (1.27–9.45)	0.015 *	0.88 (0.48–1.63)	0.683	0.79 (0.62–1.1)	0.052
	Highest	Ref	–	Ref	–	Ref	–

* Statistically significant at *p* < 0.05.

## Data Availability

The data presented in this study are available on request from the corresponding author.

## Supplementary Materials

The following supporting information can be downloaded at: https://www.mdpi.com/article/10.3390/nu15204470/s1. Table S1: Information criterion values for the logistic and mixed-effect logistic regression analyses for factors associated with stunting among under-five children, thinness among the 5–19-year group, and obesity among adults (19–59 years) using the data from urban and rural areas in South India. Table S2: Factors associated with stunting among under-five children, thinness among the 5–19 year-group, and obesity among adults (19–59 years) through a univariate mixed-effect logistic regression analysis using the data from urban and rural areas in South India.
