# Supplementary material for: Chronic Illness, Nutritional Status, and Factors Associated with Malnutrition among Various Age Groups Residing in Urban Areas of Telangana and Rural Areas of Andhra Pradesh
Source: Nutrients. 2023 Oct 22;15(20):4470. doi: 10.3390/nu15204470 (PMC10610153; doi:10.3390/nu15204470)
Supplement: Supplementary file 1 [file nutrients-15-04470-s001.zip › nutrients-2634495-supplementary.pdf]

Table-S1: Information criterion values for the logistic and mixed effect logistic regression analysis for factors associated with stunting among under-five children, thinness among 5-19 years, obesity among adults (19-59 years) using the data from urban and rural areas in south India

	Stunting		Thinness		Obesity	
	**AIC**	**BIC**	**AIC**	**BIC**	**AIC**	**BIC**
**Logistic regression**	652.5	720.4	1114.1	1181.9	4542.2	4683.4
**Mixed effect logistic regression**	643.5	715.7	1094.2	1166.9	4508.2	4655.5

Table-S2: Factors associated with stunting among under-five children, thinness among 5-19 years, obesity among adults (19-59 years) through a univariate mixed-effect logistic regression analysis using the data from urban and rural areas in south India

Variable	Category	Under 5 children			p-value	5 - 19 Years			p-value	Adult (>19&<60 years)			p-value
		Stunting		OR (95% CI)		Thinness		OR (95% CI)		Obese		OR (95% CI)	
		Yes (N=174) n (%)	No (N=343) n (%)			Yes (N=277) n (%)	No (N=669) n (%)			Yes (N=1630) n (%)	No (N=1807) n (%)		
**Place of residence**	Urban	143 (82.2)	280 (81.6)	1.09 (0.52 – 2.29)	0.827	254 (91.7)	547 (81.8)	3.43 (1.72 – 6.84)	<0.001*	1235 (75.8)	1356 (75)	1.03 (0.84 – 1.26)	0.782
	Rural	31 (17.8)	63 (18.4)	Ref	–	23 (8.3)	122 (18.2)	Ref	–	395 (24.2)	451 (25)	Ref	–
**Age group in years**	0 – 2	89 (51.1)	132 (38.5)	2.06 (1.19 – 3.57)	0.010*	–	–	–	–	–	–	–	–
	3 – 5	85 (48.9)	211 (61.5)	Ref	–	–	–	–	–	–	–	–	–
	5 – 9	–	–	–	–	81 (29.2)	183 (27.4)	Ref	–	–	–	–	–
	10 – 14	–	–	–	–	95 (34.3)	213 (31.8)	1.00 (0.61 - 1.66)	0.985	–	–	–	–
	15 – 19	–	–	–	–	101 (36.5)	273 (40.8)	0.84 (0.51 - 1.39)	0.497	–	–	–	–
	20 – 39	–	–	–	–	–	–	–	–	865 (53.1)	1212 (67.1)	Ref	–
	40 – 59	–	–	–	–	–	–	–	–	765 (46.9)	595 (32.9)	2.09 (1.75 - 2.48)	<0.001*
**Gender**	Male	95 (54.6)	172 (50.1)	1.40 (0.79 - 2.47)	0.245	188 (67.9)	321 (48)	2.73 (1.83 - 4.08)	<0.001*	521 (32)	663 (36.7)	0.78 (0.66 - 0.92)	0.003*
	Female	79 (45.4)	171 (49.9)	Ref	–	89 (32.1)	348 (52)	Ref	–	1108 (68)	1142 (63.3)	Ref	–

**Religion**	Hindu	139 (79.9)	259 (75.7)	Ref	–	225 (81.5)	557 (83.3)	Ref	–	1327 (81.5)	1555 (86.2)	Ref	–
	Muslim	19 (10.9)	54 (15.8)	0.50 (0.20 - 1.23)	0.133	36 (13.1)	73 (10.9)	1.41 (0.71 - 2.78)	0.321	203 (12.5)	151 (8.4)	1.81 (1.36 - 2.42)	<0.001*
	Christianity	16 (9.2)	29 (8.5)	1.29 (0.45 - 3.73)	0.641	15 (5.4)	39 (5.8)	0.89 (0.35 - 2.27)	0.810	99 (6.1)	97 (5.4)	1.21 (0.83 - 1.76)	0.330
**House ownership**	Own	103 (59.2)	225 (65.6)	Ref	–	172 (62.1)	448 (67)	Ref	–	1202 (73.7)	1212 (67.1)	Ref	–
	Rented/leased/others	71 (40.8)	118 (34.4)	1.53 (0.84 - 2.81)	0.166	105 (37.9)	221 (33)	1.33 (0.84 - 2.10)	0.220	428 (26.3)	595 (32.9)	0.68 (0.56 - 0.82)	<0.001*
**Type of family**	Nuclear	111 (63.8)	187 (54.5)	Ref	–	213 (76.9)	491 (73.4)	Ref	–	1106 (67.9)	1265 (70)	Ref	–
	Extended/three generation family	48 (27.6)	123 (35.9)	0.54 (0.28 - 1.04)	0.065	60 (21.7)	160 (23.9)	0.83 (0.49 - 1.40)	0.486	408 (25)	441 (24.4)	1.07 (0.87 - 1.31)	0.526
	Joint	15 (8.6)	33 (9.6)	0.65 (0.23 - 1.84)	0.413	4 (1.4)	18 (2.7)	0.40 (0.08 - 1.92)	0.251	116 (7.1)	101 (5.6)	1.37 (0.94 – 2.00)	0.104
**Type of house**	Pucca	105 (60.3)	251 (73.2)	Ref	–	173 (62.5)	416 (62.2)	Ref	–	1157 (71)	1190 (65.9)	Ref	–
	Mixed	61 (35.1)	84 (24.5)	2.48 (1.21 - 5.07)	0.013*	100 (36.1)	234 (35)	1.08 (0.68 - 1.71)	0.746	443 (27.2)	557 (30.8)	0.79 (0.65 – 0.96)	0.017*
	Kutcha	8 (4.6)	8 (2.3)	4.28 (0.81 - 22.49)	0.086	4 (1.4)	19 (2.8)	0.37 (0.07 - 1.91)	0.234	30 (1.8)	60 (3.3)	0.43 (0.24 - 0.75)	0.003*
**Overcrowdin g**	Not overcrowded	49 (28.2)	121 (35.3)	Ref	–	74 (26.7)	243 (36.3)	Ref	–	908 (55.7)	875 (48.4)	Ref	–
	Overcrowded	125 (71.8)	222 (64.7)	1.67 (0.88 - 3.16)	0.114	203 (73.3)	426 (63.7)	1.93 (1.19 - 3.12)	0.008*	722 (44.3)	932 (51.6)	0.71 (0.60 - 0.84)	<0.001*
**Place of cooking**	Separate kitchen	82 (47.1)	182 (53.1)	Ref	–	139 (50.2)	337 (50.4)	Ref	–	859 (52.7)	807 (44.7)	Ref	–
	No separate kitchen	92 (52.9)	161 (46.9)	1.48 (0.82 - 2.67)	0.193	138 (49.8)	332 (49.6)	1.03 (0.67 - 1.59)	0.899	771 (47.3)	1000 (55.3)	0.68 (0.58 - 0.81)	<0.001*
**Cooking fuel**	Gas	165 (94.8)	334 (97.4)	Ref	–	271 (97.8)	646 (96.6)	Ref	–	1598 (98)	1719 (95.1)	Ref	–
	Firewood/others	9 (5.2)	9 (2.6)	2.91 (0.64 - 13.29)	0.168	6 (2.2)	23 (3.4)	0.60 (0.16 - 2.32)	0.463	32 (2)	88 (4.9)	0.33 (0.20 - 0.55)	<0.001*
**Source of water for drinking and cooking**	Piped water into residence/ /Buying water with cans/ Water purifier in the house	161 (92.5)	310 (90.4)	Ref	–	268 (96.8)	615 (91.9)	Ref	–	1442 (88.5)	1559 (86.3)	Ref	–
	Public tap /Public well/bore/well on residence/plot	13 (7.5)	33 (9.6)	0.55 (0.18 - 1.65)	0.283	9 (3.2)	54 (8.1)	0.29 (0.11 - 0.80)	0.016*	188 (11.5)	248 (13.7)	0.80 (0.61 – 1.04)	0.090

**Most common method of defecation**	Use Toilet at Home	139 (79.9)	275 (80.2)	Ref	–	256 (92.4)	618 (92.4)	Ref	–	1549 (95)	1664 (92.1)	Ref	–
	Public / Community/Shared Toilet	5 (2.9)	10 (2.9)	0.98 (0.19 – 5.02)	0.981	10 (3.6)	20 (3)	1.23 (0.35 - 4.29)	0.748	33 (2)	47 (2.6)	0.74 (0.42 - 1.30)	0.296
	Open Field	30 (17.2)	58 (16.9)	0.98 (0.47 – 2.04)	0.949	11 (4)	31 (4.6)	1.01 (0.36 - 2.86)	0.982	48 (2.9)	96 (5.3)	0.47 (0.30 - 0.74)	0.001*
**Method of garbage disposal**	Panchayat/corporation garbage disposal services/Garbage dump/public pits	150 (86.2)	298 (86.9)	Ref	–	262 (94.6)	593 (88.6)	Ref	–	1394 (85.5)	1510 (83.6)	Ref	–
	Drainage/ Vacant/abandoned house/ Burning	13 (7.5)	23 (6.7)	1.22 (0.39 - 3.80)	0.726	5 (1.8)	37 (5.6)	0.22 (0.06 - 0.77)	0.018*	158 (9.7)	164 (9.1)	1.04 (0.77 - 1.40)	0.791
	No designated place	11 (6.3)	22 (6.4)	0.93 (0.29 - 2.95)	0.896	10 (3.6)	39 (5.8)	0.53 (0.19 - 1.49)	0.230	78 (4.8)	133 (7.4)	0.59 (0.41 - 0.85)	0.005*
**Occupation code**	Professional/ Semi-professional	–	–	–	–	–	1 (0.1)	–	–	43 (2.6)	50 (2.8)	1.09 (0.63 - 1.87)	0.767
	Clerk/ Skilled works/ Semi-skilled works	–	–	–	–	9 (3.2)	23 (3.4)	0.26 (0.02 - 3.40)	0.307	1259 (77.2)	1203 (66.6)	1.44 (1.13 - 1.84)	0.003*
	Unskilled worker	–	–	–	–	3 (1.2)	3 (0.4)	Ref	–	193 (11.8)	262 (14.5)	Ref	–
	Unemployed	–	–	–	–	2 (0.7)	7 (1.1)	0.16 (0.01 - 3.93)	0.262	43 (2.6)	81 (4.5)	0.69 (0.42 - 1.13)	0.140
	Student/Preschool	–	–	–	–	263 (94.9)	635 (95)	0.25 (0.02 - 2.56)	0.241	92 (5.6)	211 (11.7)	0.47 (0.33 - 0.69)	<0.001*
**Literate (age> 7 years)**	Illiterate	–	–	–	–	–	–	–	–	377 (23.1)	395 (21.9)	Ref	–
	Read	–	–	–	–	–	–	–	–	14 (0.9)	13 (0.7)	1.07 (0.42 - 2.76)	0.888
	Read & Write	–	–	–	–	–	–	–	–	1237 (75.9)	1398 (77.4)	0.86 (0.71 – 1.04)	0.129
	Preschool/Balwadi School	–	–	–	–	–	–	–	–	2 (0.1)	1 (0.1)	2.46 (0.14 - 43.57)	0.539
**Wealth Index**	Lowest	101 (58)	148 (43.1)	4.10 (1.83 - 9.17)	0.001*	148 (53.4)	317 (47.3)	1.43 (0.84 - 2.43)	0.185	587 (36)	880 (48.7)	0.45 (0.37 - 0.55)	<0.001*
	Middle	42 (24.2)	75 (21.9)	3.23 (1.33 - 7.87)	0.010*	61 (22.1)	169 (25.3)	0.94 (0.50 - 1.74)	0.833	393 (24.1)	424 (23.5)	0.67 (0.53 - 0.84)	0.001*
	Highest	31 (17.8)	120 (35)	Ref	–	68 (24.5)	183 (27.4)	Ref	–	650 (39.9)	503 (27.8)	Ref	–

***Significant at P – value < 0.05.**
